# Evaluating the efficacy of therapeutic HIV vaccines through analytical treatment interruptions

**DOI:** 10.7448/IAS.18.1.20497

**Published:** 2015-11-09

**Authors:** Gina M Graziani, Jonathan B Angel

**Affiliations:** 1 Ottawa Hospital Research Institute, Ottawa, ON, Canada; 2 Division of Infectious Disease, The Ottawa Hospital, Ottawa, ON, Canada

**Keywords:** HIV, AIDS, analytical treatment interruption(s), clinical trials, outcome measure, therapeutic vaccine, vaccine efficacy, viral reservoir

## Abstract

**Introduction:**

The development of an effective therapeutic HIV vaccine that induces immunologic control of viral replication, thereby eliminating or reducing the need for antiretroviral therapy (ART), would be of great value. Besides the obvious challenges of developing a therapeutic vaccine that would generate effective, sustained anti-HIV immunity in infected individuals is the issue of how to best assess the efficacy of vaccine candidates.

**Discussion:**

This review discusses the various outcome measures assessed in therapeutic HIV vaccine clinical trials involving individuals receiving suppressive ART, with a particular focus on the role of analytical treatment interruption (ATI) as a way to assess the virologic control induced by an immunotherapy. This strategy is critical given that there are otherwise no readily available measures to determine the ability of a vaccine-induced immune response to effectively control HIV replication. The various outcome measures that have been used to assess vaccine efficacy in published therapeutic HIV vaccine clinical trials will also be discussed. Outcome measures have included the kinetics of viral rebound, the new viral set point and changes in the size of the viral reservoir. Clinically relevant outcomes such as the CD4 decline, the time to resume therapy or the time to meet the criterion to resume therapy, the proportion of participants who resume therapy and/or the development of clinical symptoms such as acute retroviral syndrome are also measures of vaccine efficacy.

**Conclusions:**

Given the lack of consistency between therapeutic HIV vaccine trials in how efficacy is assessed, comparing vaccines has been difficult. It would, therefore, be beneficial to determine the most clinically relevant measure for use in future studies. Other recommendations for future clinical trials also include studying compartments in addition to blood and replacing ATIs with single-copy assays in situations in which the use of an ATI is not ideal.

## Introduction

The idea that HIV-positive individuals might benefit from therapeutic immunization was first proposed by Jonas Salk in 1987 [1]. The discovery since then of long-term non-progressors and elite controllers whose immune systems naturally control HIV infection without the need for antiretroviral therapy (ART) provides evidence for effective host-mediated anti-HIV immunity, thus providing a rationale for the development of therapeutic vaccines (reviewed in Refs. [2–4]).

The development of an HIV therapeutic vaccine capable of inducing control of HIV replication such that ART could be eliminated is a major focus of HIV research [5–7]. While ART has transformed HIV infection into a chronic, manageable disease for most individuals who have access to it [8,9], ART is associated with a number of disadvantages and limitations. In addition to being a lifelong therapy [7,8,10], ART can be toxic [8,9], is potentially associated with the development of HIV drug resistance [9] and does not eliminate latent HIV in viral reservoirs [6–9]. Finally, the high cost of ART makes it unavailable to the majority of the world's HIV-positive individuals who live in resource-limited countries [8–11]. A therapeutic vaccine would, therefore, circumvent many of the limitations associated with ART.

Besides the obvious challenges of developing a therapeutic vaccine that would induce effective, sustained anti-HIV immunity in infected individuals is the issue of how to best assess the efficacy of vaccine candidates [12]. In many clinical trials of therapeutic HIV vaccines (Tables 1A–1E), assessing efficacy involves comparing various outcome measures before and after an analytical treatment interruption (ATI), which is used to assess vaccine-induced, immune-mediated viral control [2,5]. While therapeutic HIV vaccine clinical trials typically include the CD4 count as a safety/clinical event, virologic outcome measures vary from trial to trial, making it challenging to compare the results of different vaccine studies.

**Table 1A:** Summary of the outcome measures of efficacy assessed in therapeutic HIV vaccine clinical trials with analytical treatment interruptions: protein or peptide subunit vaccines

Vaccine	Study design	Primary outcome measure(s)	Other relevant efficacy outcome measures	Main findings	References
Vacc-4x (a mixture of four p24-like peptides)	Open, prospective RCT comparing low vaccine dose vs. high vaccine dose (no ATI during this phase of study)	Safety	CD4 T cell count CD8 T cell count	The higher dose of the vaccine induced stronger HIV-specific DTH and CD4 and CD8 T cell responses than the lower dose.	[136]
	Observation period of 26 weeks following immunization period in Ref. [136] that included two ATIs, one of four weeks’ duration and one of 14 weeks	Viral load ratio (end of study viral load/pre-ART viral load set point) Immunogenicity	CD4 T cell count CD8 T cell count	Participants with the highest DTH responses before ATI had lower VL by the end of the study compared to participants with low DTH responses.	[137]
	Long-term observation (1.5 years) after immunization in Ref. [136]	Percentage of participants who resumed ART Immunogenicity	CD4 T cell count pVL	Participants with the greatest DTH responses following immunization were less likely to require ART resumption compared to low responders.	[138]
	Observation period four years after enrolling in Ref. [136]	Time until ART resumption Immunogenicity	CD4 T cell count CD8 T cell count pVL Percentage of participants who resumed ART	Participants with the greatest DTH responses following immunization resumed ART later than low responders.	[139]
	RCT	Percentage of participants who met the criteria to resume ART Percent change in CD4 T cell count between the start of the ATI and the last CD4 T cell count before ART was resumed or the end of the study if ART was not resumed	Time to restart ART CD4 T cell count CD8 T cell count Pre-ART viral load set point (when available)* Viral load set point during ATI* *substudy	The vaccine had no effect on the proportion of participants who resumed ART or on changes in the CD4 T cell count during the ATI. However, vaccinated participants had significantly reduced viral load set points during ATI compared to controls.	[111]
TUTI-16 (synthetic HIV-1 Tat epitope)	RCT	Safety	Prevention of viral rebound following ATI CD4 T cell count	The vaccine did not prevent viral rebound following ATI.	[140]
LFn-p24C (subtype C HIV Gag protein p24 fused to a detoxified anthrax-derived polypeptide)	Open label, single-arm study; phase 1A: three immunizations; phase 1B: booster + ATI	Safety	CD4 T cell count Percentage of participants who did not experience viral rebound	Immunized participants had significantly higher CD4 T cell counts compared to historical controls 12 months after enrolment in phase 1A and 30% of participants did not experience any viral rebound following ATI in phase 1B.	[141]

**Table 1B:** Summary of the outcome measures of efficacy assessed in therapeutic HIV vaccine clinical trials with analytical treatment interruptions: inactivated HIV vaccines

Vaccine	Study design	Primary outcome measure(s)	Other relevant efficacy outcome measures	Main findings	References
Remune^®^ (inactivated HIV-1 particles)	Open label, non-randomized, two-arm study (immunized vs. unimmunized)	Immunogenicity pVL	CD4 T cell count	Immunization-induced HIV-specific immune responses that correlated with CD4 T cell counts and with viral control during ATI.	[142]
Remune^®^+ART intensification with ddI, hydroxyurea and GM-CSF	Proof-of-concept, single-arm study	Immunogenicity Viral load decrease: the difference between the viral load plateaus of the first two ATIs	CD4 T cell count Percentage of CD4 T cells	Following ART intensification+Remune^®^, HIV-specific IFN-γ secretion increased between the first two of three ATIs, while viral load decreased significantly, although there was no correlation between these two observations.	[143]

**Table 1C:** Summary of the outcome measures of efficacy assessed in therapeutic HIV vaccine clinical trials with analytical treatment interruptions: DNA vaccines

Vaccine	Study design	Primary outcome measure(s)	Other relevant efficacy outcome measures	Main findings	References
DNA vaccine encoding the HIV-1 Nef, Rev and Tat proteins	RCT	Changes in immune responses of previously immunized HIV-positive participants following ART initiation.	CD4 T cell count CD8 T cell count pVL	In a substudy in which participants had undergone ATI, there was no significant change in HIV-specific responses during or after ATI.	[144]
VRC-HIV DNA 009-00-VP (a four-plasmid mixture encoding modified envelope constructs from HIV-1 subtypes A, B and C and a subtype B Gag-Pol-Nef fusion protein)	RCT; participants initiated ART during early HIV infection	Safety Immunogenicity	CD4 T cell count Viral load set point following ATI	The vaccine was safe but not immunogenic and had no effect on the viral set point during ATI.	[104]
DNA vaccine consisting of seven plasmids encoding HIV-1 Gag (subtypes A and B), Env (subtypes A, B or C), RT or Rev	RCT	HIV-specific epitope reactivity Immunogenicity Time to ART resumption	CD4 T cell count Viral load rebound following ATI Viral load set point following ATI	Although immunogenic, the vaccine did not affect the viral set point during ATI or the time to resume ART.	[145]

**Table 1D:** Summary of the outcome measures of efficacy assessed in therapeutic HIV vaccine clinical trials with analytical treatment interruptions: viral vectors

Vaccine	Study design	Primary outcome measure(s)	Other relevant efficacy outcome measures	Main findings	References
MVA-Nef (modified vaccinia Ankara virus encoding the HIV-1 LAI Nef gene)	Single-arm study	Safety Immunogenicity	Time to viral rebound following ATI Time to peak viremia following ATI Peak viremia following ATI Number of participants who resumed ART	The vaccine was safe and immunogenic but did not prevent viral rebound during ATI. However, in the majority of participants, viral load during ATI and CD4 T cell counts were improved compared to pre-ART levels.	[146]
MVA.HIVA (modified vaccinia Ankara virus encoding clade A HIV-1 Gag p24/p17 and a multi-CTL epitope)	Extension of a single-arm study by Dorrell *et al*. [147]; in this extension study, participants were boosted then underwent an ATI	IL-10 production Immunogenicity	Criteria for ART resumption (pVL and CD4 T cell count)	Vaccination did not increase IL-10 levels. However, IL-10 levels did increase during ATI and were correlated with pVL.	[148]
MVA-B (modified vaccinia Ankara virus encoding monomeric gp120 and the clade B fused Gag-Pol-Nef polyprotein)±disulfiram	RCT	Safety and immunogenicity	Kinetics of viral load rebound following ATI Time and criteria to resume ART Cell-associated HIV-1 RNA HIV-1 proviral DNA levels	The vaccine was safe and immunogenic but did not significantly affect viral load rebound after ATI or the size of the viral reservoir, whether given alone or with disulfiram.	[149]
ALVAC-HIV vCP1452 (a recombinant canarypox virus encoding HIV-1 Env, Gag and protease and part of the Nef and RT proteins)	RCT	pVL at the end of the ATI	CD4 T cell count Percentage of CD4 T cells Kinetics of viral load rebound Viral load set point following ATI	ATI, but not vaccination, contributed to enhanced viral control.	[150]
	RCT	Immunogenicity	Time to resume ART (viral rebound >50 000 copies/ml following ATI) CD4 T cell count HIV-1 DNA in PBMCs	Although immunogenic, the vaccine-induced immune responses were associated with reduced time to resume ART and greater viral rebound.	[151]
	RCT	Safety Immunogenicity Viral load set point during ATI	CD4 T cell count Percentage of CD4 T cells	The mean viral load set point during ATI did not differ between the two vaccine groups (second vaccine group received autologous DC loaded with ALVAC vCP1452).	[152]
ALVAC vCP1452 + rgp160	Two-arm study (vaccinated participants from a previous study vs. unvaccinated participants); participants initiated ART during early HIV infection	Time to viral rebound after ATI Initial rate of viral rebound after ATI Peak viremia during ATI	CD4 T cell count	ATI was followed by viral rebound in all subjects and was not affected by vaccination.	[105]
ALVAC-HIV vCP1452±IL-2	RCT	pVL at Weeks 11 and 12 post-ATI	Viral load set-point during ATI Peak viral load during ATI CD4 T cell count CD8 T cell count Disease progression, opportunistic infections or acute retroviral syndrome after ATI	Immunization with ALVAC resulted in a statistically significant reduction in viral rebound following ATI. The addition of IL-2 to ALVAC increased CD4 T cell counts but did not further reduce viral rebound.	[153]
ALVAC-HIV vCP1452±Remune^®^	RCT; participants initiated ART during acute HIV infection	Percentage of participants with pVL≤1000 HIV-1 RNA copies/ml at 24 weeks post-ATI	CD4 T cell count CD8 T cell count Cell-associated HIV-1 DNA and RNA Viral load set point during ATI Percentage of participants with pVL ≤400 HIV-1 RNA copies/ml during entire ATI period Time to reach pVL > 1000 HIV-1 RNA copies/ml after ATI	Although immunogenic, the vaccines did not induce virologic control during ATI.	[106]
	RCT	Time to viral rebound >50 HIV-1 RNA copies/ml	Safety CD4 T cell count Viral load 12 weeks after ATI Viral load set point following ATI Time to ART resumption Time to meet criteria to resume ART	ALVAC±Remune^®^ was associated with an increased time to meet the predefined criteria to restart ART and tended to delay viral rebound, but did not reduce the viral set point during ATI.	[120]
	Viral reservoir substudy of Ref. [120]	Size of the viral reservoir	CD4 T cell count	ALVAC±Remune^®^ did not affect the size of the viral reservoir.	[154]
ALVAC-HIV vCP1433 (a recombinant canarypox virus encoding part of HIV-1 Env, Gag, protease and multiple immunodominant Nef and Pol CTL epitopes)	Single-arm study	Percentage of participants who remained off ART 44 weeks after the initiation of the ATI among those having at least one HIV-specific T cell response during the vaccination period	CD4 T cell count Percentage of participants who resumed ART (pVL > 50,000 copies/ml within eight weeks of ATI or two consecutive measurements > 10,000 copies/ml any time after eight weeks of ATI) CD4 and/or CD8 HIV-specific immune responses	11% of the participants with at least one HIV-specific T cell response during vaccination remained off ART 44 weeks after the initiation of ATI.	[155]
ALVAC-HIV vCP1433 + HIV Lipo6-Tfollowed by three cycles of IL-2. The Lipo-6T vaccine is a mixture of the tetanus toxoid TT-830–843 class II restricted universal CD4 T cell epitope and five HIV-1_LAI_ peptides: Gag 17–35, Gag 235–284, Nef 66–97, Nef 116–145 and Pol 325–355.	RCT	Percentage of participants who responded to both HIV p24 and at least one of 11 HIV peptides	CD4 T cell count HIV-1 DNA in PBMC HIV-specific CD8 T cell responses (IFN-γ production) Percentage of participants experiencing virologic success following ATI Viral load set point during ATI Time to virologic failure	The vaccines induced both HIV-specific CD4 and CD8 T cell responses. Vaccine-induced immune responses predicted virologic control during ATI.	[109]
	RCT; participants initiated ART during acute HIV infection	Percentage of participants with a CD4 T cell response to HIV p24 or to one HIV peptide at Week 36	HIV-specific CD8 T cell responses Percentage of participants with virologic success at study end Time without ART HIV-1 DNA in PBMC CD4 T cell count	Vaccination did not induce CD4 T cell immune responses, had a transient impact on CD8 T cell IFN-γ responses and had no effect on viral rebound during ATI.	[107]
rFPV vaccines (recombinant fowlpox virus that encodes HIV Gag/Pol±human IFN-γ)	Extension study of an RCT by Emery *et al*. [156] in which participants who had initiated ART during acute HIV infection received placebo or rFPV±human IFN-γ; in this extension study, participants received a booster and then underwent ATI one week later.	Time-weighted mean area-under-the-curve change from baseline log pVL until ART resumption	Kinetics and rate of pVL rebound Median time to ART resumption	Immunization with rFPV Gag/Pol + IFN-γ, but not with rFPV Gag/Pol or placebo, was associated with a trend toward reduced plasma viral load following ATI.	[108]
Replication-defective adenovirus 5 HIV-1 Gag	RCT	Time averaged area-under-the curve analysis of pVL during ATI Viral load set point after ATI	CD4 T cell count	The vaccine did not significantly affect viral rebound kinetics during ATI.	[157]
	Follow-up study of Ref. [157]	pVL set point (mean of the ATI weeks 12 and 16 pVL)	Immunogenicity	The majority of the initial viral suppressors had been vaccinated; this suppression was transient.	[158]
	Retrospective analysis of Ref. [157]	Cell-associated HIV-1 DNA and RNA Residual viremia (SCA)	Immunogenicity	Vaccination had a modest, transient impact on residual viremia.	[159]

**Table 1E:** Summary of the outcome measures of efficacy assessed in therapeutic HIV vaccine clinical trials with analytical treatment interruptions: autologous dendritic cell vaccines

Vaccine	Study design	Primary outcome measure(s)	Other relevant efficacy outcome measures	Main findings	References
Autologous monocyte-derived dendritic cells loaded with heat-inactivated autologous HIV-1	RCT	Safety Percentage of participants with a set point pVL decrease of 0.5 log10 HIV-1 RNA copies/ml after the second ATI	Dynamics of pVL rebound after the second ATI compared to the first ATI CD4 T cell count CD8 T cell count CD4/CD8 ratio	Vaccination resulted in transient, partial virologic control.	[160]
	RCT	Safety Change in pVL set point during ATI (Week 24) Percentage of participants with a decrease in pVL ≥ 1 log10 at Week 24	pVL set point changes at Weeks 12, 36 and 48 (during ATI) Percentage of participants with a decrease in pVL≥1 log10 at Weeks 12, 36 and 48 (during ATI) Percentage of participants with a decrease in pVL≥0.5 log10 at Weeks 12, 24, 36 and 48 (during ATI) Percentage of participants who restarted ART CD4 T cell count	Vaccination resulted in a significant but transient reduction in viral load during ATI, which was associated with increased HIV-1-specific T cell responses.	[110]
	Viral reservoir substudy of Ref. [110]	Total and integrated HIV-1 DNA in CD4 T cells	Immunogenicity CD4 T cell count CD8 T cell count	Vaccination had no effect on the size of the viral reservoir during the vaccination period, although vaccine-induced T cell responses transiently delayed the replenishment of the viral reservoir after ATI.	[112]
Autologous monocyte-derived DCs loaded with seven HIV-1-derived CTL epitope peptides	Single-arm study	Safety Immunogenicity	CD4 T cell count Serum HIV-1 RNA Viral load rebound after ATI Viral load set point during ATI	Vaccination was safe and immunogenic in some participants but did not reduce the viral load set point during ATI.	[161]
ANRS HIV-LIPO-5 (autologous monocyte-derived DCs loaded with five HIV-1-antigen peptides [Gag(17–35), Gag(253–284), Nef(66–97), Nef(116–145) and Pol(325–355)], each covalently linked to a palmitoyl-lysylamide moiety)	Single-arm study	Safety	ART resumption CD4 T cell count Serious non-AIDS events AIDS-defining events Maximum viral load during ATI	The vaccine was safe and induced HIV- specific CD4 T cell responses that were associated with a trend toward reduced maximum viral load during ATI.	[162]
Autologous monocyte-derived DCs loaded with ALVAC-HIV vCP1452	RCT	Safety Immunogenicity Viral load set point during ATI	CD4 T cell count Percentage of CD4 T cells	The mean viral load set point during ATI did not differ between the two vaccine groups (another vaccine group received ALVAC-HIV vCP1452 independently of DC).	[152]
Autologous monocyte-derived DCs loaded with mRNA encoding HIV-1 Tat, Rev and Nef	Single-arm study	Safety	CD4 T cell count CD8 T cell count Kinetics of viral rebound during ATI Duration off ART	The vaccine was safe and immunogenic. Although 6/17 participants remained off ART 96 weeks post-ATI, there was no correlation between HIV-specific immune responses and time off ART.	[163]

RCT: randomized controlled trial; ATI: analytical treatment interruption; VL: viral load; ART: antiretroviral therapy; pVL: plasma viral load; IFN-γ: IFN-gamma; ddI: didanosine; GM-CSF: granulocyte-macrophage colony-stimulating factor; Nef: HIV negative regulatory factor; Rev: HIV regulator of expression of virion proteins; Tat: HIV transactivator of transcription; Gag: HIV group antigens; Pol: HIV precursor of reverse transcriptase, protease and integrase; Env: HIV envelope; RT: HIV reverse transcriptase; DCs: dendritic cells; IL-2: interleukin-2; rFPV: recombinant fowlpox virus; IL-10: interleukin-10; CTL: cytotoxic T lymphocytes; PBMC: peripheral blood mononuclear cells; SCA: single-copy assay.

## Discussion

### The current state of non-HIV therapeutic vaccines

Only a few therapeutic vaccines are currently licensed worldwide and most of them are used to treat cancer [13]. In 2010, the US Food and Drug Administration (FDA) approved sipuleucel-T (Provenge^®^) to treat hormone-refractory prostate cancer [14]. A therapeutic vaccine for ovarian cancer has been approved in Dubai [13], while another one was recently given fast track designation by the FDA [15]. Two different therapeutic vaccines for renal cell carcinoma have been approved, one each in Russia and South Korea [13]. In the meantime, phase III clinical trials have been or are being conducted to assess the efficacy of candidate therapeutic vaccines against a variety of malignancies including cancers of the breast [16,17], pancreas [18–20], liver [21], lung [22–27], kidneys [28], skin [29–32], prostate [33], stomach or oesophagus [34] and brain [35–38].

Zostavax^®^ is a therapeutic vaccine that reduces the frequency and severity of shingles, which is caused by the reactivation of the varicella zoster virus that causes chickenpox [39]. Zostavax is the first example of a vaccine with clinical efficacy against an established infection [40]. The success of this vaccine provides hope that it might be possible to induce clinically beneficial immunity against other viruses that establish chronic infections.

The development of therapeutic vaccines to treat other chronic infections in humans is an area of active research. The various pathogens, other than HIV, against which therapeutic vaccines are currently being or have been assessed in various clinical trials include cytomegalovirus [41–43], hepatitis B [44–61] and hepatitis C [62–76] viruses, human papillomavirus [77–90], herpes simplex virus 2 [91–93], *Mycobacterium tuberculosis* [94,95], *Trypanosoma cruzi* [96] and *Leishmania* [97]. Encouraging results have been obtained in some of these trials [43,47,48,59,63–66,68,76,79–82,84,86].

### The current state of therapeutic HIV vaccines assessed in clinical trials

The features that might make a therapeutic vaccine effective and the inherent challenges of making such a vaccine have been recently described in a number of excellent reviews [3,6,98–102]. Minimally, a therapeutic vaccine should improve the benefits of existing ART regimens, simplify these regimens or allow for periodic ART interruption [6,98–100]. Ideally, a therapeutic vaccine would eliminate the need for ART, either by eradicating virus (a sterilizing cure) or by inducing an immune response capable of controlling virus replication (a functional cure) [6,98–100,102].

Therapeutic vaccination would be of particular value for HIV-positive individuals residing in resource-limited countries in which access to ART is limited [98]. In these settings, the HIV epidemic is fuelled by the higher rate of new infections relative to the rate at which newly infected individuals receive ART [98,99]. An effective therapeutic vaccination could, therefore, help control the epidemic. Therapeutic vaccines would also be invaluable to those who struggle with daily, lifelong ART compliance [98].

Over the course of more than two decades, more than four dozen therapeutic HIV vaccine candidates have been evaluated in clinical trials for safety, immunogenicity and, in some cases, for efficacy. The results of these trials have shown limited success (reviewed in Refs. [3,6,99–102]) with respect to their ability to control HIV replication or maintain CD4 T cell counts in the absence of ART [99,102,103]. While the majority of these trials involved therapeutic vaccination of individuals who initiated ART during chronic HIV infection, vaccination of individuals who initiated ART during acute or early infection was also ineffective [104–108]. In one of these studies, the dynamics of viral rebound following vaccination and ATI were similar to those observed in studies of chronically infected individuals who discontinued ART [105].

A few randomized, controlled clinical trials by Levy *et al*. [109], Garcia *et al*. [110] and Pollard *et al*. [111] have, however, produced somewhat encouraging results.

In a trial assessing the effects of receiving two vaccines, ALVAC-HIV vCP1433 and Lipo6-T, followed by IL-2 administration, Levy *et al*. [109] observed that a significantly greater proportion of vaccinated HIV-positive participants had a lower viral set point 12 weeks after stopping ART, compared to unvaccinated controls. The times to viral rebound and to resume therapy were also significantly delayed in the vaccinated participants.

Garcia *et al*. [110] observed that therapeutic vaccination of HIV-positive participants with an autologous dendritic cell vaccine loaded with autologous, inactivated HIV-1 resulted in a decrease in the viral load set point following ATI. Unfortunately, the decrease in the viral load induced by vaccination was transient. Furthermore, vaccination did not prevent the CD4 T cell count decline after interruption of ART. It was subsequently reported that, although no change was observed in the size of the viral reservoir during the vaccination period, vaccine-induced T cell responses transiently delayed the replenishment of the viral reservoir after ATI [112].

Pollard *et al*. [111] observed that the Vacc-4x vaccine, which contains a mixture of conserved Gag peptide domains, was able to significantly reduce the viral load following ATI, resulting in a new viral load set point. However, vaccination did not affect the change in the CD4 T cell count following ATI, nor did it affect the proportion of participants who resumed therapy or the time until therapy was resumed.

### The role of ATIs in assessing the efficacy of therapeutic HIV vaccines

ART may be interrupted as part of a structured treatment interruption (STI) or as part of an ATI. The main goals of the STI are to reduce ART-associated burden (reviewed in Refs. [4,113–115]) and/or to induce HIV “autoimmunization” (reviewed in Refs. [3,4,114,116]), whereas the purpose of the ATI is to assess the efficacy of an experimental therapeutic candidate [12]. STIs and ATIs are discussed in further detail below.

STIs have been used in the past to address the ART-associated issues of toxicity, cost and resistance (reviewed in Refs. [4,113–115]). Another goal of the STI was to allow for viral rebound, resulting in “autoimmunization” with increased exposure to HIV antigens (reviewed in Refs. [3,4,114,116]). It was hypothesized that the resulting viremia would boost the anti-HIV immune response sufficiently to induce viral control, thus avoiding ART resumption. Unfortunately, the various clinical trials that assessed the immunological benefits of STIs failed to show any benefits (reviewed in Refs. [4,114]), while the SMART study showed that treatment interruptions can increase morbidity and mortality [117].

When ART is interrupted, plasma HIV RNA levels typically first become detectable within days or weeks [118–120], reach a peak and then decrease to a steady state level, or viral set point [121]. Exceptions to the occurrence of viral rebound following therapy interruption do occur and may be more frequent in those who are treated during acute primary infection [122], although the exact immune mechanisms responsible for this degree of viral control are currently unknown.

The ATI is an intentional interruption of ART that is included in controlled clinical trials of therapeutic vaccines (reviewed in Refs. [2,4,5,115,123]). The ATI is a frequently used strategy for assessing HIV therapeutic vaccine efficacy [12]; it is considered by some to be the “gold standard” [5]. This strategy, which is used to assess the virologic control induced by an immunotherapy given while the individual is still taking suppressive ART [2,115,123] is necessary because there are currently no laboratory assays that measure the ability of the immune system to effectively control HIV replication [2,99,123]. In addition to assessing the kinetics of viral rebound [2,99], ATIs also allow for the assessment of a potentially new viral set point as well as CD4 T cell dynamics following treatment interruption [115,123].

The SMART study revealed that HIV-positive participants who interrupted ART experienced an increased risk of developing AIDS and non-AIDS events compared to participants who continued therapy [117]. However, it also showed that individuals having high CD4 counts (>500 cells/µl), high CD4 nadir (>200 cells/µl) and undetectable virus levels (<50 copies/ml) can safely undergo treatment interruptions in carefully monitored clinical trials without increasing their risk of death and non-AIDS events or developing viral resistance [115,124–126]. It was recently shown that chronically infected individuals having undetectable virus levels and preserved CD4 counts, including those with low CD4 nadir, can also safely undergo treatment interruptions if the interruptions are short, that is if treatment is reinitiated upon detection of viral rebound [127].

Despite the safety of ATIs, the increased viral load that occurs following treatment interruption can occasionally be associated with the development of an acute retroviral syndrome [128,129] or thrombocytopenia [130], as well as with an increased risk of HIV transmission by individuals involved in high-risk activities [131].

### Alternatives to ATIs

In studies that include an ATI, therapy is typically reintroduced either at the end of a fixed period of treatment interruption (e.g. 16 weeks), during which time a new viral set point is usually achieved, or when specific virologic, immunologic or clinical outcomes are met. A new, alternative treatment interruption strategy in clinical trials of HIV immunotherapies is the monitored antiretroviral pause (MAP), which reintroduces ART as soon as viral rebound occurs [123]. The advantage of the MAP is that, by reintroducing ART as soon as viral rebound occurs, the risk is reduced compared to the risk associated with an ATI. However, since the MAP does not allow a new viral set point to be established, this strategy cannot be used to determine whether the immunotherapy being tested improved virologic control by the immune system. Thus, whether an ATI or a MAP should be used in a clinical trial of an HIV immunotherapy depends on the scientific question being asked, with the MAP lending itself to assess therapies designed to measure the time to viral rebound, which may be a surrogate measure of the size of the viral reservoir, while the ATI should be used to assess therapies designed to improve immune control of HIV. It should be noted, however, that it has not yet been established whether the time to viral rebound following ATI is, in fact, a surrogate measure of the size of the viral reservoir [132].

Recently, single-copy reverse transcriptase (RT)-qPCR assays with single-copy sensitivity (i.e. the single-copy assay (SCA) for HIV-1 RNA) were used to detect virus in the plasma of individuals who had undergone myeloablation and autologous stem cell transplantation for the treatment of lymphoma [133]. Since these patients had undetectable plasma viremia by standard methods, it was hypothesized that their lymphoma treatment had resulted in HIV eradication; the results of the SCA, however, proved otherwise. Therefore, in this setting, SCA was used to guide the decision regarding whether to interrupt ART; because virus was detected using this assay, ART was not interrupted and the viral rebound that would have otherwise occurred was avoided. However, had the SCA failed to detect virus, then an ATI would have been warranted. The use of these highly sensitive assays has been suggested as an additional approach to the assessment of therapeutic vaccine efficacy [5]. The inclusion of such assays into future clinical trials of HIV immunotherapeutics could expedite these trials if only subjects with undetectable viral load by SCA proceeded on to ATI. However, such an alternative approach would need to be validated first by concurrent analysis in clinical trials in which it can be determined that SCA results predict virologic rebound following ATI [5]. In the interim, or until some other strategy is validated, treatment interruptions, the current gold standard for assessing therapeutic vaccine efficacy [5], will continue to play a crucial role in the evaluation of HIV therapeutic vaccines and should only be replaced with some other strategy if treatment interruption must be avoided.

It was recently shown that the *ex vivo* antiviral capacity of CD8+ T cells [134] predicts the rate of CD4 T cell loss in early HIV infection and is inversely correlated with viral load set point [135]. It has been suggested, therefore, that this assay [134] be included as a read-out in clinical trials of therapeutic vaccines. However, whether this accurately measures vaccine-induced immunologic control of viral replication remains to be established [135].

### Read-outs of therapeutic HIV vaccine studies that incorporate ATIs

More than four dozen therapeutic HIV vaccine candidates have been evaluated in clinical trials for safety, immunogenicity and, in some cases, for efficacy. Tables 1A–1E summarizes the outcome measures of efficacy that have been assessed in published vaccine trials that include ATI.

Since the correlates of viral suppression/immunological response that should be used to assess the therapeutic benefits of vaccines are not well defined [7,99,135,164–166], the surrogate measure(s) used as the primary end point(s) to assess the clinical benefits of therapeutic vaccine candidates vary from trial to trial.

The virologic outcome measures assessed following vaccination and ATI may include the time to detectable viremia, the peak level of viremia, the new viral set point, the time to reach the new viral set point, the time to reach a certain viral load threshold, the viral load at a predefined time following ATI and changes in the size of the viral reservoir.

Of these read-outs, it has been suggested that the new viral set point is the most relevant clinical assessment of the antiviral efficacy of a therapeutic vaccine (reviewed in Ref. [5]). Whereas the new viral set point, the peak level of viremia and the time to rebound are all affected by the strength of the host's anti-HIV immune response, the peak level of viremia and the time to rebound may also be affected by the number of susceptible target cells and the size of the viral reservoir, respectively. It has also been suggested that the new viral set point established after immunotherapy and ATI should be the primary end point of clinical trials for assessing the effectiveness of anti-HIV immunotherapies; a difference of at least 0.5 log copies/ml between the experimental and control arms of a study is probably clinically significant, as determined by the results obtained in studies of antiretrovirals (reviewed in Ref. [2]). One disadvantage of using the new viral set point as a primary end point, however, is that it may miss important virologic and immunologic events that occur early in the immune response to the vaccine [165]. Another disadvantage is that the establishment of a new viral set point may be delayed, thus extending the length of treatment interruption and its associated risks.

Another primary end point that is commonly used in HIV therapeutic vaccine clinical trials is the time to detectable virus or the time to viral rebound (i.e. the time to achieve a viral load >50 copies/ml) following an ATI. However, an accurate assessment of this requires frequent viral load monitoring [165]. While this outcome would seem to be clinically relevant, its value is unknown since this measure does not appear to correlate with other virologic outcome measures. In a therapeutic vaccine trial of ALVAC-HIV vCP1452 by Jacobson *et al*., the time until viral rebound did not correlate with any of the other virologic measures assessed, such as the new viral load set point [150]. As a result, it has been suggested that the time to viral rebound is probably not an appropriate outcome measure for assessing the effectiveness of HIV therapeutic vaccines [2]. Similarly, our own study of ALVAC-HIV vCP1452 with or without Remune^®^ failed to find a correlation between the time to viral rebound and the new viral set point, nor with the magnitude of the viral rebound [120]. A correlation was observed, however, between the time to viral rebound and the time to restart therapy, as well as the time to meet the criteria to do so. In the one other trial in which vaccination was found to delay the time to viral rebound (this trial involved ALVAC-HIV vCP1433 and Lipo-6T), no assessments were made for correlations with other virologic outcome measures [109].

The CD4 count, which is routinely used to determine the risk of opportunistic infection [167], is typically included in trials of HIV therapeutic vaccines. In addition to monitoring the change in the absolute CD4 count, changes in the percentage of CD4 T cells, the CD4:CD8 ratio, the time to decline to a predefined level or the change in the slope of the CD4 count have also been assessed in clinical trials of HIV therapeutic vaccines. However, this is not an ideal primary outcome as it requires waiting for a decline in the CD4 count.

In addition to virologic and immunologic outcomes, some studies of therapeutic HIV vaccines include the assessment of clinical outcomes. These outcomes include the development of clinical events, including symptoms of acute retroviral syndrome after ATI [128,129] or the time until either ART is resumed or the criteria for ART resumption is met. In addition, the proportion of participants who do or do not resume ART may also be determined.

### Therapeutic HIV vaccines and their potential role in an HIV cure strategy

One of the research priorities recently identified by the International AIDS Society (IAS) Global Scientific Strategy “Towards an HIV Cure” working group is the development of a therapeutic HIV vaccine capable of boosting the immune system of the infected host to control HIV replication in the absence of ART, thus producing a functional cure [7] similar to that experienced naturally by long-term non-progressors and elite controllers (reviewed in Refs. [7,99,166]). According to this IAS working group, a therapeutic vaccine should be directed to conserved HIV epitopes and either 1) elicit a humoral response consisting of neutralizing anti-HIV antibodies that would a) prevent cell-to-cell transmission or b) recognize virus-producing cells for destruction by antibody-dependent cellular cytotoxicity; or 2) induce a strong cytotoxic cellular response for the destruction of cells producing virus before virus progeny is released [7]. These strategies should lead to a sustained reduction in the number of cells actively transcribing virus and induce an immune selective pressure that would lead to loss of viral fitness and replicative potential.

The persistence of the viral reservoir is considered to be the major obstacle to curing HIV infection [3,6,7,10]. In fact, when ART is interrupted, the viral rebound that occurs within days or weeks is the result of reseeding from viral reservoirs [11]. Furthermore, high levels of HIV DNA, a surrogate marker of the size of the viral reservoir, are correlated with quicker viral rebound following ART interruption [126]. The few trials of therapeutic vaccines that have assessed the change in the size of the viral reservoir did not observe any significant effect [106,112,149,154,159,168–172]. Five of these studies assessed whether vaccination induced any changes in the size of the viral reservoir by measuring proviral DNA, either by co-culture assay [154,168,171] or by PCR [149,169]. One of the advantages of assessing changes in the size of the viral reservoir as a read-out of therapeutic vaccine efficacy is that this outcome measure can be made in trials that do not include an ATI [168], thus minimizing the risks that may be associated with treatment interruptions. Disadvantages of using this outcome measure, however, include the fact that no single assay accurately measures the size of the viral reservoir [173,174] and the lack of strong correlations between assays [174].

## Conclusions and future directions

The development of a therapeutic HIV vaccine would be a valuable alternative to the use of expensive, toxic, lifelong ART regimens. The results of dozens of clinical trials performed over more than two decades to assess the safety, immunogenicity and, in many cases, the efficacy of various HIV therapeutic vaccines have been published, and more trials are underway. Besides the obvious challenges of developing a successful therapeutic vaccine is the issue of how to best assess the efficacy of vaccine candidates [12]. Currently, the inconsistent assessment of different outcome measures in different trials makes it difficult to compare the relative efficacies of the various vaccine candidates.

In its “Towards an HIV Cure” recommendations, the IAS recommends that future clinical trials of therapeutic HIV include studying compartments in addition to peripheral blood, such as the gastrointestinal tract and lymph nodes, both sites of HIV infection and immune responses [7]. Other suggestions for future trials include measuring immune control of viral replication by using highly sensitive SCA in situations in which the use of ATI is not ideal.

Given that immune activation predicts HIV disease progression independently of the CD4 count and viral load, it has also been recommended that, when assessing the efficacy of an HIV immunotherapy such as therapeutic vaccination, concurrent changes in immune activation markers, vaccine-specific responses and viral replication should be assessed during treatment interruption [175].

It is apparent that despite significant efforts made, the therapeutic vaccine candidates studied to date have been associated with limited clinical benefit [3,6,99]. Continued efforts will be required, therefore, to develop and test a safe and effective therapeutic HIV vaccine that will help end the global HIV epidemic. Future work may be influenced by promising prophylactic simian immunodeficiency virus (SIV) vaccines. In one study, virus levels became undetectable following initial viremia in half of the macaques vaccinated prior to SIV challenge [176], while in another SIV/macaque study, one-third of the monkeys that became infected following SIV challenge ultimately became elite controllers [177]. Thus, despite being designed as preventative SIV vaccines, both appeared to induce therapeutic benefits. These simian vaccines may, therefore, provide some valuable insight into the design of effective therapeutic HIV vaccines.

Finally, while the characteristics of a successful therapeutic HIV vaccine are currently unknown, standardizing which outcome measures should be used in future clinical trials to evaluate vaccine efficacy would certainly be beneficial.

## Author information

JBA is the Co-leader of the Vaccines and Immunotherapies (VIT) Core of the CTN (CIHR Canadian HIV Trials Network). GMG is the VIT Core Research Associate.
